# Carbohydrate-Rich Diet Is Associated with Increased Risk of Incident Chronic Kidney Disease in Non-Diabetic Subjects

**DOI:** 10.3390/jcm8060793

**Published:** 2019-06-04

**Authors:** Ki Heon Nam, Seong Yeong An, Young Su Joo, Sangmi Lee, Hae-Ryong Yun, Jong Hyun Jhee, Seung Hyeok Han, Tae-Hyun Yoo, Shin-Wook Kang, Jung Tak Park

**Affiliations:** 1 Department of Internal Medicine, College of Medicine, Institute of Kidney Disease Research, Yonsei University, Seoul 03722, Korea; khheon@yuhs.ac (K.H.N.); jys8427@gmail.com (Y.S.J.); leesm2332@yuhs.ac (S.L.); siberian82@yuhs.ac (H.-R.Y.); hansh@yuhs.ac (S.H.H.); yoosy0316@yuhs.ac (T.-H.Y.); kswkidney@yuhs.ac (S.-W.K.); 2 Division of Integrated Medicine, Department of Internal Medicine, College of Medicine, Yonsei University, Seoul 03722, Korea; 3 Department of Internal Medicine, Dongkang Medical Center, Ulsan 44455, Korea; genemy@naver.com; 4 Division of Nephrology and Hypertension, Department of Internal Medicine, Inha University College of Medicine, Incheon 22332, Korea; jjhlove77@yuhs.ac; 5 Department of Internal Medicine, College of Medicine, Severance Biomedical Science Institute, Brain Korea 21 PLUS, Yonsei University, Seoul 03722, Korea

**Keywords:** dietary carbohydrate, carbohydrate density, chronic kidney disease, renal nutrition

## Abstract

Despite the potential relationship with metabolic derangements, the association between dietary carbohydrate intake and renal function remains unknown. The present study investigated the impact of dietary carbohydrate intake on the development of incident chronic kidney disease (CKD) in a large-scale prospective cohort with normal renal function. A total of 6746 and 1058 subjects without and with diabetes mellitus (DM) were analyzed, respectively. Carbohydrate intake was assessed by a 24-h dietary recall food frequency questionnaire. The primary endpoint was CKD development, defined as a composite of estimated glomerular filtration rate (eGFR) of ≤60 mL/min/1.73 m^2^ and the development of proteinuria. CKD newly developed in 20.1% and 36.0% of subjects during median follow-ups of 140 and 119 months in the non-DM and DM subjects, respectively. Categorization of non-DM subjects into dietary carbohydrate density quartiles revealed a significantly higher risk of CKD development in the third and fourth quartiles than in the first quartile (*P* = 0.037 for first vs. third; *P* = 0.001 for first vs. fourth). A significant risk elevation was also found with increased carbohydrate density when carbohydrate density was treated as a continuous variable (*P* = 0.008). However, there was no significant difference in the incident CKD risk among those with DM according to dietary carbohydrate density quartiles. Carbohydrate-rich diets may increase the risk of CKD development in non-DM subjects.

## 1. Introduction

Chronic kidney disease (CKD) is a major public health burden worldwide due to its close association with the development of end-stage renal disease, cardiovascular disease (CVD), and premature death [1]. Despite blood glucose control and hypertension therapy, which are key factors affecting kidney function, the prevalence of CKD continues to rapidly increase [2]. Because established CKD is irrevocable, identifying novel and modifiable risk factors is meaningful for the provision of preventive strategies to reduce morbidity and mortality.

Dietary carbohydrate intake has been repeatedly reported to have an impact on metabolic disorders. High dietary carbohydrate consumption has been found to have adverse effects on lipid and glucose metabolism, leading to insulin resistance and obesity [3,4,5,6,7,8,9,10,11]. In addition, a low carbohydrate diet has been noted as an effective weight loss strategy for obese individuals [12]. Considering that metabolic risk factors such as diabetes and obesity are closely linked with an increased CKD risk [13,14,15,16], it is plausible to surmise that the amount of dietary carbohydrate may also affect kidney function. However, the impact of dietary carbohydrates on kidney function has not been well evaluated. Although a recent investigation has evaluated the effect of dietary carbohydrate in overweight and obese individuals, impact in the general population is not known [17,18]. In addition, due to the highly controlled diet setting and relatively short observation duration of that study, the results are difficult to generalize in real-world settings.

Therefore, the present study evaluated the association between dietary carbohydrate intake and incident CKD development, defined as a composite of estimated glomerular filtration rate (eGFR) of ≤60 mL/min/1.73 m^2^ and the development of proteinuria, in a prospective community-based cohort of subjects with normal renal function. Carbohydrate intake was assessed by a 24-h dietary recall food frequency questionnaire (FFQ). The dietary density of carbohydrate intake was defined as the percentage of energy (% E) provided by carbohydrates, which was computed by dividing energy from the carbohydrates by the total daily energy intake.

## 2. Materials and Methods

### 2.1. Study Design and Participants

All subjects were recruited from the Korean Genome Epidemiology Study (KoGES) launched in 2001 by the Korean government (National Research Institute of Health, Centers for Disease Control and Prevention, and Ministry of Health and Welfare). The study rationale, design, methods, and protocol summary are described in detail elsewhere [19]. Briefly, residents aged between 40 and 69 years from urban (Ansan) and rural (Ansung) areas in Korea were initially recruited. A total of 10,030 participants who voluntarily provided informed consents were enrolled in the study. The participants underwent medical examinations and various surveys at baseline. 

**Figure 1:**
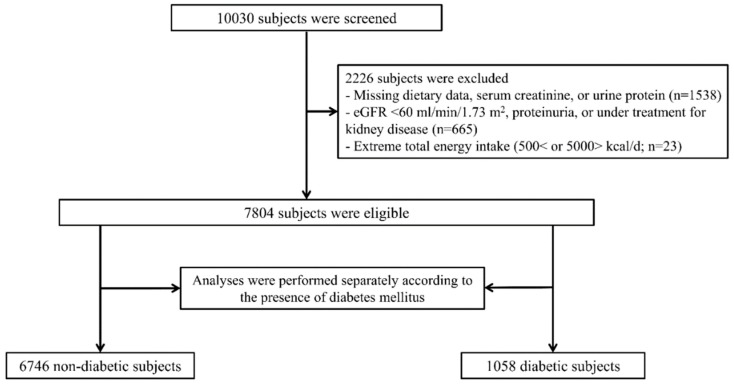
Flow diagram of the study cohort. (Abbreviation: eGFR, estimated glomerular filtration rate).

Serial follow-up assessments were performed biennially until 2014. Subjects with missing data, underlying kidney disease, or with extreme total energy intake (<500 or >5000 kcal/day) were excluded. A total of 7804 subjects were included in the final analysis. All analyses were conducted separately according to the presence of diabetes mellitus (DM) due to the possibility for dietary carbohydrate to differently effect outcome based on the presence of underlying DM (Figure 1). The study was performed in accordance with the Declaration of Helsinki and approved by the Institutional Review Board of Yonsei University Health System Clinical Trial Center (4-2016-0100).

### 2.2. Data Collection and Measurements

At the time of study entry, all participants responded to a standardized self-administered questionnaire on health and lifestyle. The participants were questioned by trained interviewers regarding their demographic and socioeconomic data including age, sex, level of education, income, marital status, lifestyle (i.e., smoking habits, alcohol consumption, daily physical activity), reproductive history, psychological stress, social relationships and disease history (i.e., disease status of the participants and his/her family members). For dietary assessment, a semi-quantitative FFQ involving 103 items was developed for the KoGES. Education status was categorized into three groups: low (lower than middle school), middle (middle school), and high (higher than middle school). Income status was also categorized into three groups: low (<$850 per month), middle (>$850 to <$1700 per month), and high (>$1700 per month). Daily physical activities were expressed as metabolic equivalents of task. Subjects with a medical history of hypertension, with a blood pressure (BP) of >140/90 mm Hg, or receiving antihypertensive treatment were considered hypertensive. Those with a medical history of DM, blood glucose levels of ≥126 mg/dL after an eight-hour fast, post-load glucose levels of ≥200 mg/dL after a 75-g oral glucose tolerance test, hemoglobin A1c (HbA1c) ≥6.5%, or taking medication for hyperglycemia were considered to have DM. Subjects with a medical history of dyslipidemia or taking lipid-lowering agents were considered to have dyslipidemia. CVD was defined as the composite of coronary artery disease, myocardial infarction, congestive heart failure, peripheral artery disease, and/or cerebrovascular disease. The subjects underwent anthropometric measurements performed by trained healthcare providers. The body mass index and waist-to-hip ratio (WHR) were calculated as the weight divided by squared height (kg/m^2^) and the waist circumference divided by the hip circumference, respectively. BP was measured in a seated position after subjects had been in a relaxed state for at least 10 min.

Bio-specimens included fasting blood samples that were collected in a serum separator tube and two ethylenediaminetetraacetic acid tubes, and a 10-mL midstream urine sample [19]. The following biochemical data were measured: concentrations of blood urea nitrogen, creatinine, glucose, HbA1c, insulin, albumin, total cholesterol, triglyceride, high-density lipoprotein cholesterol (HDL-C), C-reactive protein, hemoglobin, and white blood cell count. The low-density lipoprotein cholesterol (LDL-C) concentration was calculated using Friedewald’s equation [20]. Insulin resistance was assessed using the homeostasis model assessment of insulin resistance (HOMA-IR) equation (fasting insulin [µIU/mL] × fasting glucose [mg/dL]/405) [21]. The eGFRs was calculated using the CKD-epidemiology collaboration equation [22]. Urinalysis was conducted on fresh urine samples measured semiquantitatively by urine dipstick (URISCAN Pro II; YD Diagnostics Corp., Seoul, Korea) and reported as one of five grades: absent, trace, 1+, 2+, or 3+, corresponding to protein levels of <10, 10–20, >30, >100, and >500 mg/dL, respectively. Proteinuria was defined as a grade of 1+ or greater. 

### 2.3. Dietary Intake Measurements

Single-day dietary data for total calorie (kcal), carbohydrate (g), protein (g), and fat (g) intakes were assessed using a semi-quantitative dietary recall FFQ, which was surveyed by trained interviewers at baseline and four years after the initial FFQ. The details of the FFQ have been described and its validity and reproducibility have been verified previously [23]. The questionnaire comprises a food list of nine frequency-based intake items and three items regarding intake amounts. Each participant was asked to report the frequency and amount of food they had consumed on average over the past year. Data were inputted into the cohort epidemiology information system designed to calculate nutrition and food intake for each participant, which analyzed each connected item through a nutritional database. Two food frequency questionnaire assessments were conducted during the study period, one at baseline and another at four-years follow-up. The average values of the FFQs conducted at the four-year interval were used for the analysis. The total dietary nutrient and calorie components were calculated using the 2011 nutrient database of the Korean Nutrition Society. The dietary density of nutrient intake (carbohydrate, protein, and fat) was defined as the percentage of energy (% E) provided by nutrients, which was computed by dividing energy from the nutrient by the total daily energy intake.

### 2.4. Study Endpoint

The primary outcome was incident CKD, defined as a composite of eGFR <60 mL/min/1.73 m^2^ and/or the development of proteinuria.

### 2.5. Statistical Analyses

Continuous variables were expressed as means ± standard deviation if data were normally distributed and as medians (interquartile ranges) if the distributions were skewed. Categorical variables were expressed as numbers and proportion. Comparisons were made using one-way analysis of variance (ANOVA) or Kruskal-Wallis tests for continuous variables and chi-square tests for categorical variables. The normality of the distributions was ascertained by Kolmogorov-Smirnov tests. As mentioned above, the patients were first divided into two groups according to the presence of DM and were further stratified into quartiles based on the dietary carbohydrate density. For trend analysis, Jonckheere-Terpstra tests and linear-by-linear association were used for continuous variables and categorical variables, respectively. The tendency of the variables across the dietary carbohydrate density quartiles were reported based on results of trend analyses. The time to development of incident CKD was estimated by the Kaplan-Meier method and statistical differences between groups according to dietary carbohydrate density were compared by log-rank tests. Patients lost to follow-up were censored at the date of the last examination. Cox proportional hazards regression analyses were constructed to determine the independent predictive value of dietary carbohydrate intake on development of incident CKD. Variables that showed statistical significance in univariate analyses (Supplementary Table S1) and variables that were known to have a clinical implication on CKD development were selected as covariates in multivariate analyses. Multivariate Cox hazards models were constructed with three incremental levels of adjustment: (i) Model 1: unadjusted; (ii) Model 2, adjusted for age, sex, and baseline eGFR; (iii) Model 3, further adjusted for WHR, education status, marital status, smoking status, exercise, hypertension, and cardiovascular disease; (iv) Model 4, laboratory parameters including hemoglobin, HOMA-IR, albumin, HDL-C, and triglyceride were further added. The results of these Cox models are presented as hazard ratios (HRs) and 95% confidence intervals (CIs). Changes in participants’ metabolic profiles throughout the follow-up period were analyzed with repeated measures ANOVA. For all analyses, statistical significance was defined as *P* < 0.05. Data were analyzed using IBM SPSS Statistics for Windows, version 23.0 (IBM Corp., Armonk, NY, USA) and Stata version 14.2 (Stata Corp., College Station, TX, USA).

## 3. Results

### 3.1. Baseline Characteristics

A flowchart of participants is shown in Figure 1. The baseline nutritional and sociodemographic characteristics of the non-DM and DM subjects are shown in Table 1; Table 2, respectively. Their mean ages were 51.3 ± 8.6 and 55.4 ± 8.8 years, and there were 3182 (47.2%) and 556 (52.5%) male subjects. The mean (range) dietary carbohydrate density values were 71.5 ± 6.0% (36.6–87.2%) in non-DM subjects and 71.9 ± 6.1% (47.8–87.6%) in DM subjects. The baseline laboratory characteristics of the non-DM and DM subjects are shown in Table 3 and Table 4. The mean of eGFRs were 93.2 ± 13.0 and 90.3 ± 13.1 mL/min/1.73 m^2^, respectively.

In both non-DM and DM groups, subjects with higher dietary carbohydrate density revealed significantly lower total energy intake, protein density, and fat density. Participants in the higher carbohydrate density quartiles among both non-DM and DM subjects tended to be female (*P* < 0.001, trend analysis), less educated (*P* < 0.001, trend analysis), have lower income (*P* < 0.001, trend analysis), and were less likely to be married (*P* < 0.001, trend analysis). They were also more likely to be non-smokers, non-drinkers, and physically more active (*P* < 0.001, trend analysis). The systolic BPs and WHRs were also elevated in those with higher dietary carbohydrate density, regardless of DM status. The proportions of subjects with a history of hypertension or CVD significantly increased across the dietary carbohydrate density quartiles in non-DM but not in DM subjects.

Laboratory data assessments showed that eGFR levels were comparable among the carbohydrate density quartiles regardless of DM status. Non-DM and DM subjects in the higher carbohydrate density groups tended to have lower levels of hemoglobin (*P* < 0.001, trend analysis), albumin (*P* < 0.001, trend analysis), glucose (*P* < 0.001, trend analysis), total cholesterol (*P* < 0.001 for non-DM; *P* = 0.002 for DM; trend analysis), and LDL-C (*P* < 0.001 for non-DM; *P* = 0.018 for DM; trend analysis). The levels of HDL-C significantly decreased (*P* < 0.001, trend analysis), whereas the HOMA-IR (*P* < 0.001, trend analysis) and triglyceride values (*P* < 0.001, trend analysis) tended to increase in non-DM subjects with higher dietary carbohydrate density quartiles. However, there were no intergroup differences in HOMA-IR, triglyceride, and HDL-C levels in subjects with DM.

### 3.2. Development of Incident CKD

During a median (range) follow-up duration of 140.0 (93.4–143.1) months in subjects without DM and 118.7 (47.7–42.0) months in those with DM, CKD developed in 1359 (20.1%) and 381 (36.0%) subjects, respectively.

### 3.3. Metabolic Profiles According to Dietary Carbohydrate Density

**Figure 2:**
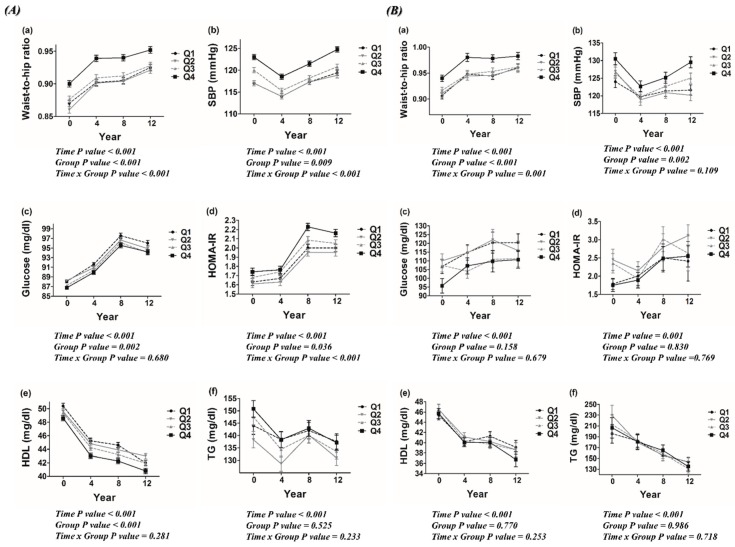
Changes of metabolic parameters according to dietary carbohydrate density in (**A**) non-DM and (**B**) DM subjects. (Abbreviations: SBP, systolic blood pressure; HOMA-IR, homeostasis model assessment of insulin resistance; HDL-C, high-density lipoprotein cholesterol; TG, triglyceride).

Among non-DM subjects, WHR, systolic BP, and HOMA-IR levels were higher and HDL-C levels were lower in the quartile with the highest dietary carbohydrate density throughout the study period (Figure 2A). Among DM subjects, WHR and systolic BP showed similar patterns to those in subjects without DM. However, HOMA-IR and HDL-C levels were comparable among the dietary carbohydrate density quartiles during the follow-up period (Figure 2B).

### 3.4. Risk of Incident CKD According to Dietary Carbohydrate Intake

**Figure 3:**
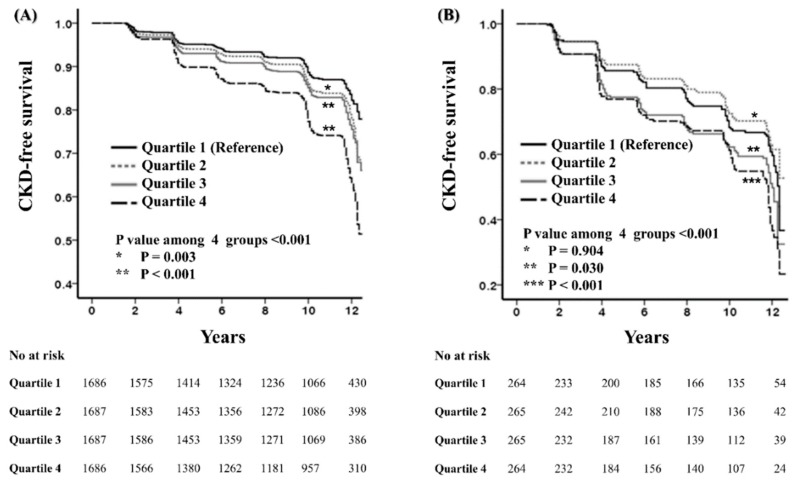
Cumulative curves of CKD-free survival according to dietary carbohydrate density in non-DM (**A**) and DM (**B**) subjects. (Abbreviations: CKD, chronic kidney disease; DM, diabetes mellitus).

The Kaplan–Meier survival curves of cumulative survival free from incident CKD according to dietary carbohydrate density are shown in Figure 3. In the non-DM group, compared with the first carbohydrate density quartile, the time to CKD development was shorter in the quartiles with higher carbohydrate density (*P* = 0.003 for first vs. second; *P* < 0.001 for first vs. third or fourth) (Figure 3A). Among subjects with DM, the time to CKD development was significantly shorter in the third and fourth quartiles than in the first carbohydrate density quartile (*P* = 0.03 for first vs. third; *P* < 0.001 for first vs. fourth) (Figure 3B).

To assess the risk of incident CKD, multivariate Cox hazards models were constructed. In non-DM subjects, the risk of CKD development was significantly higher in the third (71.9–75.8%; HR, 1.20; 95% CI, 1.01–1.43; *P* = 0.04) and fourth (>75.8%; HR, 1.35; 95% CI 1.13–1.60; *P* = 0.001) quartiles than in the first dietary carbohydrate density quartile (<67.8%). When dietary carbohydrate density was treated as a continuous variable, a significant association between it and incident CKD was also found (HR for every 10% increase, 1.15; 95% CI, 1.04−1.28; *P* = 0.008). This relationship was independent of confounding metabolic factors. However, there was no significant difference in the incident CKD risk among participants with DM assigned to each dietary carbohydrate density quartile (Table 5).

## 4. Discussion

In this study, higher dietary carbohydrate density was significantly associated with an increased risk of CKD development among non-DM participants with normal renal function. In addition, this elevation in incident CKD risk gradually increased with higher amounts of dietary carbohydrate density. However, such significant relationship between dietary carbohydrate and incident CKD was not found in subjects with DM.

Reports on carbohydrate intake and kidney function are few and the results are often controversial. A randomized control trial of obese adults showed that a low-carbohydrate diet did not affect kidney function [24]. Meanwhile, a meta-analysis of eGFR from nine randomized control trials evaluating the effect of low-carbohydrate diet on weight loss revealed increased eGFR in both low-carbohydrate and control diet groups [17]. However, the eGFR increase was greater with a low-carbohydrate diet than control diet, suggesting a beneficial effect of lower dietary carbohydrate levels. Nevertheless, the greater eGFR increase with low carbohydrate diet in that study could be related to hyperfiltration since low carbohydrate diets are often associated with higher protein intake [25]. In contrast, a study of older adults observed an increased risk of CKD only for energy-dense, nutrition-poor carbohydrate intake and not for other carbohydrate-containing food groups [26]. The present observational study of over 10 years showed a significantly increased risk of CKD development in non-DM individuals with higher dietary carbohydrate density. The larger number of participants and longer observation duration could account for the differences from previous studies. In addition, the effect of carbohydrate density differences among diets from diverse cultural backgrounds may also have played a role. Generally, Asian foods have a relatively higher carbohydrate density than that of western meals [27]. In the current study the average carbohydrate density was around 70%, a relatively higher level compared to previously reported carbohydrate densities of around 50% in western diets [27]. 

Several mechanisms could be speculated for the association between dietary carbohydrate and incident CKD. As metabolic derangements such as insulin resistance and dyslipidemia are known risk factors for CKD [13,14,15,16], metabolic abnormalities accompanied with higher dietary carbohydrate density may play key mechanistic roles. In this study, WHR and HOMA-IR had a significant time group interaction, indicating worsening metabolic impairment with time in those whose diets contained more carbohydrates among non-DM subjects supporting the possibility of a dietary carbohydrate, metabolic abnormality, CKD link. However, still other causal factors should also be considered since the association between high dietary carbohydrate density and incident CKD remained significant even after adjusting for metabolic abnormality components. One of these factors may be systemic inflammation. Increased intake of high-glycemic index carbohydrates has been found to activate nuclear factor-kappaB in young healthy subjects [28]. In addition, short-term acute hyperglycemia caused by refined carbohydrate intake increased circulating levels of free radicals and proinflammatory cytokines such as interleukin (IL)-6, IL-18, and tumor necrosis factor-alpha [29]. Oxidative stress could also have played a role. Increased dietary glycemic load has been reported to be closely related to greater oxidative stress, represented by elevated levels of lipid peroxidation markers such as malondialdehyde and F2-isoprostanes [30,31]. Recently, deregulation of gut microbiota has emerged as a factor affecting renal function [32,33]. Accumulating evidences have shown that dietary changes alter microbial composition [34]. High dietary levels of glucose or fructose resulted in a loss of gut microbial diversity and increased intestinal permeability and subsequent development of metabolic disorders in mice [35]. Based on these results, it is feasible that diet related gut microbiota alteration could also affect renal function. Further studies would be needed to delineate the linkage between high carbohydrate diets, gut microbiota, and the development of CKD.

High dietary carbohydrate density was associated with increased CKD risk in non-DM subjects only. A previous randomized study showed that clinical markers of renal function did not differ between individuals with type 2 DM consuming high and low carbohydrate diets [36]. In addition, a recent meta-analysis reported that high or low carbohydrate diets did not significantly affect metabolic markers in patients with type 2 DM [37]. These reports are concordant with our finding that the effect of dietary carbohydrate varied depending on the presence of DM. As DM is a significant risk factor for CKD [38], the negative effect of higher dietary carbohydrates on renal function could have been diluted in the presence of DM. The fact that HOMA-IR and HDL-C was related with dietary carbohydrate density only in non-DM subjects could also be considered as one of the causes of the current finding. This possibility is plausible since insulin resistance is a well-recognized risk factor for CKD [13,16,39]. In addition, a recent investigation showing a causal association between genetically higher HDL cholesterol concentration and better kidney function provides a link between dietary carbohydrates, HDL-C, and CKD [40]. However, despite the possible intermediary role of insulin resistance and HDL-C, the relationship between dietary carbohydrate density and incident CKD persisted even after adjusting for HOMA-IR and HDL-C in the multivariable models, suggesting yet another factor related to this association. Further investigations uncovering these factors are required.

The study has several limitations. First, given the observational nature of the study design, the possible effects of unaccounted residual confounding could not be ruled out. However, to reduce this risk, vigorous adjustments were made for potential confounding factors including demographic, clinical, and laboratory parameters. Second, dietary intake was assessed by a dietary recall FFQ. Although dietary recall somewhat lacks precision, this method has been proven to provide adequate dietary intake measurements for large-scale studies. In addition, the validity and reproducibility of the FFQ used in the present study were previously verified, further supporting the reliability of the dietary data used in this study [23,41]. Third, the study population was composed exclusively of an Asian ethnicity from a single nation. Since dietary components vary among cultural and geographical regions, the findings of this study may not be generalized to other cultural backgrounds. Further research is needed to determine the impact of carbohydrate intake on renal function in diverse cultural settings.

In conclusion, high dietary carbohydrate density could increase the risk of incident CKD development in non-DM subjects with preserved renal function. This may be related to unfavorable metabolic profiles associated with higher carbohydrate intake. Additional controlled trials are needed to evaluate the effect of modifying dietary carbohydrate on renal outcomes.

## Figures and Tables

**Table 1:** Baseline nutritional and sociodemographic characteristics of non-DM subjects.

Variables	Total (*n* = 6746)	Quartiles of Dietary Carbohydrate Density				*P* ^a^	*P* ^b^
		Q1 (*n* = 1686) <67.8	Q2 (*n* = 1687) 67.8–71.9	Q3 (*n* = 1687) 71.9–75.8	Q4 (*n* = 1686) >75.8		
**Nutrients (per day)**							
Total energy intake (kcal)	1883.2 ± 518.0	2136.4 ± 587.1	1943.3 ± 453.7	1798.0 ± 429.6	1655.3 ± 460.2	<0.001	<0.001
Carbohydrate density (%)	71.5 ± 6.0	63.7 ± 3.9	69.9 ± 1.2	73.8 ± 1.1	78.6 ± 2.2	<0.001	<0.001
Protein density (%)	13.3 ± 2.0	15.5 ± 1.7	13.7 ± 1.2	12.6 ± 1.0	11.2 ± 1.1	<0.001	<0.001
Fat density (%)	14.1 ± 4.7	20.1 ± 3.1	15.4 ± 1.5	12.4 ± 1.4	8.6 ± 1.9	<0.001	<0.001
**Demographic data**							
Age (years)	51.3 ± 8.6	48.3 ± 7.4	50.0 ± 8.1	51.5 ± 8.4	55.5 ± 8.7	<0.001	<0.001
Male (%)	3182 (47.2)	973 (57.7)	895 (53.1)	767 (45.5)	547 (32.4)	<0.001	<0.001
SBP (mmHg)	120.0 ± 17.8	117.4 ± 16.4	118.1 ± 16.9	120.7 ± 18.3	123.9 ± 18.6	<0.001	<0.001
DBP (mmHg)	79.8 ± 11.3	79.2 ± 11.3	78.9 ± 11.4	80.0 ± 11.2	81.1 ± 11.3	<0.001	<0.001
Body mass index (kg/m^2^)	24.6 ± 2.9	24.5 ± 2.8	24.5 ± 2.9	24.6 ± 3.0	24.6 ± 3.0	0.471	0.175
Waist-to-hip ratio	0.88 ± 0.07	0.86 ± 0.07	0.86 ± 0.07	0.87 ± 0.07	0.90 ± 0.07	<0.001	<0.001
Education (%)						<0.001	<0.001
Low	2074 (30.9)	268 (16.0)	344 (20.4)	544 (32.4)	918 (55.0)		
Intermediate	3698 (55.1)	1069 (63.7)	1032 (61.3)	952 (56.7)	645 (38.7)		
High	935 (13.9)	340 (20.3)	307 (18.2)	183 (10.9)	105 (6.3)		
Income (%)						<0.001	<0.001
Low	2152 (32.4)	293 (17.5)	395 (23.7)	519 (31.3)	945 (57.1)		
Intermediate	1993 (30.0)	515 (30.8)	519 (31.1)	543 (32.8)	416 (20.9)		
High	2505 (37.6)	862 (51.6)	756 (45.3)	594 (35.9)	293 (17.7)		
Marriage (yes)	6130 (91.2)	1586 (94.3)	1569 (93.3)	1533 (91.1)	1442 (85.8)	<0.001	<0.001
Alcohol (%)	3647 (54.2)	1130 (67.1)	990 (59.0)	892 (52.9)	635 (37.9)	<0.001	<0.001
Smoking (%)	2691 (40.2)	842 (50.2)	749 (44.6)	637 (38.0)	463 (27.8)	<0.001	<0.001
Exercise (MET, k)	9.8 ± 6.3	9.3 ± 6.0	9.2 ± 5.8	9.8 ± 6.4	10.9 ± 6.9	<0.001	<0.001
**Comorbidities**							
Hypertension	2470 (36.6)	541 (32.2)	552 (32.7)	611 (36.2)	766 (45.4)	<0.001	<0.001
CVD	163 (2.4)	31 (1.8)	32 (1.9)	35 (2.1)	65 (3.9)	<0.001	<0.001
Dyslipidemia	148 (2.2)	39 (2.3)	42 (2.5)	40 (2.4)	27 (1.6)	0.253	0.149

^a^ ANOVA test, *post-hoc* analyses results not shown; ^b^ Trend analysis. Note: Continuous variables are expressed as means ± standard deviation or medians (interquartile range) and categorical variables as numbers (percentage). Abbreviations: DM, diabetes mellitus; SBP, systolic blood pressure; DBP, diastolic blood pressure; MET, metabolic equivalent of task; CVD, cardiovascular disease; ANOVA, analysis of variance.

**Table 2:** Baseline nutritional and sociodemographic characteristics of DM subjects.

Variables	Total (*n* = 1058)	Quartiles of Dietary Carbohydrate Density				*P* ^a^	*P* ^b^
		Q1 (*n* = 264) <68.0	Q2 (*n* = 265) 68.0–72.2	Q3 (*n* = 265) 72.2–76.3	Q4 (*n* = 264) >76.3		
**Nutrients (per day)**							
Total energy intake (kcal)	1859.9 ± 517.5	2152.0 ± 528.4	1888.0 ± 466.4	1767.2 ± 440.8	1632.5 ± 485.5	<0.001	<0.001
Carbohydrate density (%)	71.9 ± 6.1	63.8 ± 3.7	70.2 ± 1.2	74.2 ± 1.2	79.3 ± 2.2	<0.001	<0.001
Protein density (%)	13.3 ± 2.1	15.8 ± 1.8	13.8 ± 1.1	12.8 ± 1.04	11.0 ± 1.0	<0.001	<0.001
Fat density (%)	13.6 ± 4.7	19.6 ± 3.1	14.9 ± 1.5	11.7 ± 1.4	8.0 ± 1.9	<0.001	<0.001
**Demographic data**							
Age (years)	55.4 ± 8.8	52.3 ± 8.7	54.2 ± 8.3	55.9 ± 8.7	59.3 ± 7.9	<0.001	<0.001
Male (%)	556 (52.5)	187 (70.8)	173 (65.3)	121 (45.7)	75 (28.3)	<0.001	<0.001
SBP (mmHg)	127.7 ± 18.7	124.9 ± 16.9	127.2 ± 19.6	127.4 ± 19.3	131.3 ± 18.3	0.001	<0.001
DBP (mmHg)	82.9 ± 10.8	82.6 ± 10.8	82.9 ± 11.8	82.2 ± 10.5	83.8 ± 10.2	0.417	0.472
Body mass index (kg/m^2^)	25.7 ± 3.1	25.9 ± 2.9	25.5 ± 3.1	25.7 ± 3.2	25.6 ± 3.1	0.502	<0.001
Waist-to-hip ratio	0.92 ± 0.07	0.91 ± 0.06	0.90 ± 0.07	0.91 ± 0.06	0.94 ± 0.07	<0.001	<0.001
Education (%)						<0.001	<0.001
Low	435 (41.3)	54 (20.5)	83 (31.4)	123 (46.6)	175 (67.0)		
Intermediate	480 (45.6)	143 (54.4)	151 (57.2)	109 (41.3)	77 (29.5)		
High	137 (13.0)	66 (25.1)	30 (11.4)	32 (12.1)	9 (3.4)		
Income (%)						<0.001	<0.001
Low	448 (42.8)	63 (24.0)	88 (33.6)	114 (43.5)	183 (70.7)		
Intermediate	279 (26.7)	79 (30.0)	78 (29.8)	73 (27.9)	49 (18.9)		
High	319 (30.5)	1221(46.0)	96 (36.6)	75 (28.6)	27 (10.4)		
Marriage (yes)	921 (87.5)	248 (94.3)	244 (92.4)	232 (87.9)	197 (75.2)	<0.001	<0.001
Alcohol (%)	568 (53.9)	178 (67.4)	176 (66.7)	129 (48.9)	85 (32.6)	<0.001	<0.001
Smoking (%)	484 (46.3)	164 (63.3)	146 (55.3)	100 (38.2)	74 (28.4)	<0.001	<0.001
Exercise (MET, k)	9.7 ± 6.4	9.1 ± 5.9	9.0 ± 5.7	9.5 ± 6.4	11.2 ± 7.2	<0.001	<0.001
**Comorbidities**							
Hypertension	590 (55.8)	135 (51.1)	153 (57.7)	139 (52.5)	163 (61.5)	0.083	0.062
CVD	50 (4.7)	11 (4.2)	12 (4.5)	11 (4.2)	16 (6.1)	0.703	0.370
Dyslipidemia	41 (3.9)	10 (3.8)	11 (4.2)	9 (3.4)	11 (4.2)	0.964	0.950

^a^ ANOVA test, post-hoc analyses results not shown; ^b^ Trend analysis. Note: Continuous variables are expressed as means ± standard deviation or medians (interquartile range) and categorical variables as numbers (percentage). Abbreviations: DM, diabetes mellitus; SBP, systolic blood pressure; DBP, diastolic blood pressure; MET, metabolic equivalent of task; CVD, cardiovascular disease; ANOVA, analysis of variance.

**Table 3:** Baseline laboratory characteristics of non-DM subjects.

Variables	Total (*n* = 6746)	Quartiles of Dietary Carbohydrate Density				*P* ^a^	*P* ^b^
		Q1 (*n* = 1686) <67.8	Q2 (*n* = 1687) 67.8–71.9	Q3 (*n* = 1687) 71.9–75.8	Q4 (*n* = 1686) >75.8		
**Laboratory parameters**							
BUN (mg/dL)	14.2 ± 3.5	14.2 ± 3.4	14.3 ± 3.5	14.1 ± 3.6	14.0 ± 3.5	0.066	0.031
Creatinine (mg/dL)	0.83 ± 0.17	0.87 ± 0.17	0.85 ± 0.17	0.82 ± 0.16	0.78 ± 0.15	<0.001	<0.001
eGFR (mL/min/1.73 m^2^)	93.2 ± 13.0	93.6 ± 13.1	93.3 ± 13.4	93.8 ± 13.0	92.4 ± 12.3	0.100	0.032
WBC (×1000 cells/μL)	6.46 ± 1.75	6.47 ± 1.68	6.54 ± 1.75	6.41 ± 1.78	6.40 ± 1.78	0.079	0.082
Hemoglobin (g/dL)	13.6 ± 1.6	13.9 ± 1.6	13.7 ± 1.6	13.5 ± 1.6	13.2 ± 1.5	<0.001	<0.001
Glucose (mg/dL)	82.8 ± 8.5	83.6 ± 8.4	83.3 ± 8.7	82.3 ± 8.7	81.8 ± 8.2	<0.001	<0.001
HbA1c (%)	5.5 ± 0.3	5.5 ± 0.3	5.5 ± 0.4	5.6 ± 0.3	5.6 ± 0.3	<0.001	<0.001
HOMA-IR	1.6 ± 1.0	1.6 ± 0.9	1.6 ± 1.1	1.6 ± 1.0	1.7 ± 1.1	0.004	0.001
Albumin (g/dL)	4.3 ± 0.3	4.3 ± 0.3	4.3 ± 0.3	4.2 ± 0.3	4.2 ± 0.3	<0.001	<0.001
Cholesterol (mg/dL)	190 ± 33	192 ± 34	191 ± 35	188 ± 34	189 ± 33	<0.001	<0.001
Triglyceride (mg/dL)	155 ± 95	154 ± 98	150 ± 88	156 ± 100	158 ± 92	0.105	<0.001
HDL-C (mg/dL)	44.8 ± 9.9	45.7 ± 10.0	45.2 ± 9.9	44.4 ± 9.8	44.0 ± 9.6	<0.001	<0.001
LDL-C (mg/dL)	115 ± 32	116 ± 33	116 ± 32	112 ± 31	113 ± 31	<0.001	<0.001
CRP (mg/L)	0.22 (0.09, 0.31)	0.21 (0.09, 0.30)	0.21 (0.10, 0.29)	0.22 (0.09, 0.33)	0.23 (0.09, 0.31)	0.462 ^c^	0.124

^a^ ANOVA test, post-hoc analyses results not shown; ^b^ Trend analysis; ^c^ Kruskal-Wallis test. Note: Continuous variables are expressed as means ± standard deviation or medians (interquartile range) and categorical variables as numbers (percentage). Abbreviations: DM, diabetes mellitus; BUN, blood urea nitrogen; eGFR, estimated glomerular filtration rate; WBC, white blood cell; HbA1c, hemoglobin A1c; HOMA-IR, homeostasis model assessment of insulin resistance; HDL-C, high-density lipoprotein cholesterol; LDL-C, low-density lipoprotein cholesterol; CRP, C-reactive protein; ANOVA, analysis of variance.

**Table 4:** Baseline laboratory characteristics of DM subjects.

Variables	Total (*n* = 1058)	Quartiles of Dietary Carbohydrate Density				*P* ^a^	*P* ^b^
		Q1 (*n* = 264) <68.0	Q2 (*n* = 265) 68.0–72.2	Q3 (*n* = 265) 72.2–76.3	Q4 (*n* = 264) >76.3		
**Laboratory parameters**							
BUN (mg/dL)	14.6 ± 3.6	14.8 ± 3.8	15.1 ± 3.5	14.4 ± 3.5	14.2 ± 3.8	0.042	0.018
Creatinine (mg/dL)	0.84 ± 0.17	0.89 ± 0.18	0.88 ± 0.17	0.81 ± 0.16	0.77 ± 0.14	<0.001	<0.001
eGFR (mL/min/1.73 m^2^)	90.3 ± 13.1	91.2 ± 13.7	89.2 ± 13.5	91.2 ± 13.0	89.6 ± 11.9	0.155	0.423
WBC (×1000 cells/μL)	6.85 ± 1.90	6.84 ± 1.85	6.81 ± 1.79	7.10 ± 2.08	6.66 ± 1.83	0.058	0.585
Hemoglobin (g/dL)	13.9 ± 1.5	14.3 ± 1.4	14.2 ± 1.5	13.8 ± 1.5	13.1 ± 1.4	<0.001	<0.001
Glucose (mg/dL)	120.3 ± 42.3	127.2 ± 48.6	124.0 ± 43.0	118.1 ± 35.5	110.0 ± 35.2	<0.001	<0.001
HbA1c (%)	7.3 ± 1.6	7.3 ± 1.7	7.3 ± 1.6	7.4 ± 1.5	7.1 ± 1.4	0.409	0.266
HOMA-IR	2.7 ± 2.6	2.9 ± 2.2	2.6 ± 1.7	2.8 ± 4.2	2.7 ± 2.2	0.750	0.825
Albumin (g/dL)	4.3 ± 0.3	4.3 ± 0.4	4.3 ± 0.4	4.2 ± 0.3	4.2 ± 0.3	<0.001	<0.001
Cholesterol (mg/dL)	200 ± 40	205 ± 39	206 ± 41	197 ± 37	196 ± 40	<0.001	0.002
Triglyceride (mg/dL)	209 ± 143	213 ± 138	217 ± 162	208 ± 129	200 ± 140	0.597	0.248
HDL-C (mg/dL)	42.5 ± 9.4	42.2 ± 9.0	43.2 ± 9.3	41.9 ± 9.0	42.6 ± 10.2	0.460	0.931
LDL-C (mg/dL)	117 ± 36	120 ± 36	120 ± 36	115 ± 36	114 ± 36	0.073	0.018
CRP (mg/L)	0.27 (0.09, 0.31)	0.25 (0.09, 0.30)	0.28 (0.10, 0.29)	0.29 (0.09, 0.33)	0.29 (0.09, 0.31)	0.614^c^	0.210

^a^ ANOVA test, post-hoc analyses results not shown; ^b^ Trend analysis; ^c^ Kruskal-Wallis test. Note: Continuous variables are expressed as means ± standard deviation or medians (interquartile range) and categorical variables as numbers (percentage). Abbreviations: DM, diabetes mellitus; BUN, blood urea nitrogen; eGFR, estimated glomerular filtration rate; WBC, white blood cell; HbA1c, hemoglobin A1c; HOMA-IR, homeostasis model assessment of insulin resistance; HDL-C, high-density lipoprotein cholesterol; LDL-C, low-density lipoprotein cholesterol; CRP, C-reactive protein; ANOVA, analysis of variance.

**Table 5:** Cox proportional hazards regression analyses of the association between dietary carbohydrate density and incident CKD.

Carbohydrate Density (%)	Continuous ^a^	*P*	Dietary Carbohydrate Density (vs. Q1)					
			Q2	*P*	Q3	*P*	Q4	*P*
			HR (95% CI)		HR (95% CI)		HR (95% CI)	
**Non-DM**								
Model 1	1.71 (1.55, 1.88)	<0.001	1.30 (1.09, 1.54)	0.003	1.46 (1.23, 1.72)	<0.001	2.32 (1.98, 2.71)	<0.001
Model 2	1.12 (1.01, 1.25)	0.030	1.14 (0.96, 1.36)	0.134	1.19 (1.00, 1.41)	0.047	1.35 (1.14, 1.60)	0.001
Model 3	1.18 (1.06, 1.31)	0.002	1.16 (0.97, 1.38)	0.103	1.22 (1.03, 1.45)	0.024	1.40 (1.17, 1.66)	0.024
Model 4	1.15 (1.04, 1.28)	0.008	1.14 (0.06, 1.36)	0.136	1.20 (1.01, 1.43)	0.037	1.35 (1.13, 1.60)	0.001
**DM**								
Model 1	1.45 (1.22, 1.73)	<0.001	0.87 (0.64, 1.17)	0.357	1.36 (1.03, 1.80)	0.032	1.62 (1.23, 2.12)	0.001
Model 2	1.02 (0.82, 1.27)	0.839	0.76 (0.53, 1.08)	0.121	1.07 (0.77, 1.51)	0.683	0.86 (0.60, 1.24)	0.419
Model 3	1.00 (0.80, 1.26)	0.999	0.74 (0.52, 1.06)	0.096	1.12 (0.79, 1.58)	0.525	0.83 (0.57, 1.21)	0.336
Model 4	0.96 (0.76, 1.20)	0.712	0.74 (0.52, 1.05)	0.091	1.04 (0.73, 1.47)	0.830	0.78 (0.53, 1.15)	0.208

Note: ^a^ per 10% increase for dietary carbohydrate density. Model 1: Unadjusted. Model 2: Adjusted for age, sex, and baseline eGFR. Model 3: Model 2 + waist-to-hip ratio, education status, marital status, smoking status, exercise, hypertension, and cardiovascular disease. Model 4: Model 3 + hemoglobin, HOMA-IR, albumin, high-density lipoprotein cholesterol, and triglyceride. Abbreviations: CKD, chronic kidney disease; HR, hazard ratio; CI, confidence interval; DM, diabetes mellitus; eGFR, estimated glomerular filtration rate; HOMA-IR, homeostasis model assessment of insulin resistance.

## Supplementary Materials

The following are available online at https://www.mdpi.com/2077-0383/8/6/793/s1. Supplementary Table S1. Univariate Cox proportional hazard regression analyses of factors associated with incident CKD
