# Supplementary material for: Carbohydrate-Rich Diet Is Associated with Increased Risk of Incident Chronic Kidney Disease in Non-Diabetic Subjects
Source: J Clin Med. 2019 Jun 4;8(6):793. doi: 10.3390/jcm8060793 (PMC6617052; doi:10.3390/jcm8060793)
Supplement: Supplementary file 1 [file jcm-08-00793-s001.pdf]

Carbohydrate-rich diet is associated with increased risk of incident chronic kidney disease in non-diabetic subjects

Authors:

Ki Heon Nam, Seong Yeong An, Young Su Joo, Sangmi Lee, Hae-Ryong Yun, Jong Hyun Jhee, Seung Hyeok Han, Tae-Hyun Yoo, Shin-Wook Kang, and Jung Tak Park

Supplemental Material Table of Contents

Supplemental Table 1. Univariate Cox proportional hazard regression analyses of factors associated with incident CKD

Supplemental Table 1. Univariate Cox proportional hazard regression analyses of factors associated with incident CKD

	Non-DM		DM	
	**HR (95% CI)**	**P-value**	**HR (95% CI)**	**P-value**
**Demographic data**				
Age (per 1 year)	1.10 (1.09, 1.11)	<0.001	1.08 (1.07, 1.10)	<0.001
Sex (Male)	0.77 (0.70, 0.86)	<0.001	0.69 (0.57, 0.84)	<0.001
Waist-to-hip ratio (per 0.1)	1.52 (1.41, 1.64)	<0.001	1.48 (1.27, 1.72)	<0.001
Education				
Low	*Reference*		*Reference*	
Intermediate	0.48 (0.43, 0.53)	<0.001	0.55 (0.44, 0.67)	<0.001
High	0.45 (0.38, 0.55)	<0.001	0.41 (0.28, 0.58)	<0.001
Smoking				
Never	*Reference*		*Reference*	
Former or current	0.84 (0.75, 0.94)	0.003	0.74 (0.60, 0.90)	0.003
Exercise (per 10 MET)	1.02 (0.94, 1.11)	0.589	1.06 (0.91, 1.24)	0.429
Marriage (Yes)	0.55 (0.47, 0.64)	<0.001	0.62 (0.47, 0.81)	<0.001
History of hypertension (Yes)	1.80 (1.62, 2.00)	<0.001	1.63 (1.33, 2.00)	<0.001
History of CVD (Yes)	2.00 (1.52, 2.65)	<0.001	1.64 (1.07, 2.53)	0.024
**Laboratory data**				
Baseline eGFR (per 1 ml/min per 1.73m^2)	0.95 (0.94, 0.96)	<0.001	0.97 (0.96, 0.98)	<0.001
Hemoglobin (per 1 g/dl)	0.99 (0.96, 1.02)	0.572	0.92 (0.86, 0.98)	0.007
HOMA-IR (per 1)	1.10 (1.05, 1.14)	<0.001	1.03 (0.99, 1.07)	0.170
Albumin (per 1 g/dl)	0.59 (0.49, 0.70)	<0.001	0.54 (0.40, 0.72)	<0.001
HDL-C (per 10 mg/dl)	0.92 (0.88, 0.97)	0.001	0.94 (0.85, 1.03)	0.166
Triglyceride (per 10 mg/dl)	1.01 (1.01, 1.02)	<0.001	1.01 (1.00, 1.02)	0.002

Abbreviations: DM, diabetes mellitus; HR, hazard ratio; CI, confidence interval; SBP, systolic blood pressure; MET, metabolic equivalent of task; CVD, cardiovascular disease; eGFR, estimated glomerular filtration rate; HOMA-IR, homeostasis model assessment of insulin resistance; HDL-C, high-density lipoprotein cholesterol
